# Development of a TaqMan Allelic Discrimination Assay for detection of Single Nucleotides Polymorphisms associated with anti-malarial drug resistance

**DOI:** 10.1186/1475-2875-11-23

**Published:** 2012-01-20

**Authors:** Edwin Kamau, Saba Alemayehu, Karla C Feghali, LaDonna S Tolbert, Bernard Ogutu, Christian F Ockenhouse

**Affiliations:** 1 Military Malaria Research Program, Malaria Vaccine Branch, Department of Molecular Diagnostics and Genomic Studies, Walter Reed Army Institute of Research, 503 Robert Grant Ave, Silver Spring, 20 Maryland, USA; 2 Division of Entomology, Molecular Diagnostics, Walter Reed Army Institute of Research, Silver Spring, Maryland, USA; 3 Walter Reed Project, Kenya Medical Research Institute, Kisumu, Kenya

## Abstract

**Background:**

Anti-malarial drug resistance poses a threat to current global efforts towards control and elimination of malaria. Several methods are used in monitoring anti-malarial drug resistance. Molecular markers such as single nucleotide polymorphism (SNP) for example are increasingly being used to identify genetic mutations related to anti-malarial drug resistance. Several methods are currently being used in analysis of SNP associated with anti-malarial drug resistance and although each one of these methods has unique strengths and shortcoming, there is still need to improve and/or develop new methods that will close the gap found in the current methods.

**Methods:**

TaqMan Allelic Discrimination assays for detection of SNPs associated with anti-malarial drug resistance were designed for analysis on Applied Biosystems PCR platform. These assays were designed by submitting SNP sequences associated with anti-malarial drug resistance to Applied Biosystems website. Eleven SNPs associated with resistance to anti-malarial drugs were selected and tested. The performance of each SNP assay was tested by creating plasmid DNAs carrying codons of interests and analysing them for analysis. To test the sensitivity and specificity of each SNP assay, 12 clinical samples were sequenced at codons of interest and used in the analysis. Plasmid DNAs were used to establish the Limit of Detection (LoD) for each assay.

**Results:**

Data from genetic profiles of the *Plasmodium falciparum *laboratory strains and sequence data from 12 clinical samples was used as the reference method with which the performance of the SNP assays were compared to. The sensitivity and specificity of each SNP assay was establish at 100%. LoD for each assay was established at 2 GE, equivalent to less than 1 parasite/μL. SNP assays performed well in detecting mixed infection and analysis of clinical samples.

**Conclusion:**

TaqMan Allelic Discrimination assay provides a good alternative tool in detection of SNPs associated with anti-malarial drug.

## Background

Spread and emergence of old and new drug resistant parasites is threatening malaria control programmes in most malaria endemic regions [1,2]. Advances that have been made thus far will only be sustained if monitoring and reporting of anti-malarial drug resistance becomes an integral part of malaria control efforts [3]. Anti-malarial drug resistance and drug efficacy can be monitored by performing therapeutic efficacy studies, measurement of drug concentrations in blood, *in vitro *tests that monitor parasite phenotypic changes and analysis of molecular markers that monitor genetic changes. Although therapeutic efficacy studies are considered the gold standard method for determining anti-malarial drug efficacy, each of these methods presents unique strengths and weaknesses [3-6]. Recent advances in molecular information and technology in particular has positioned analysis and monitoring of genetic markers as an important alternative method for detection of drug resistance [7,8].

*Plasmodium falciparum *manifests its genetic diversity in several forms: single nucleotide polymorphisms (SNPs), microsatellite repeats, small insertions or deletions (indels) and gene duplication. Studies identifying SNPs and gene duplications associated with anti-malarial drug resistance have been exploited in evaluation of resistance to treatment [9]. SNPs in one or more genes can be attributed to cumulative effect of resistance [10,11]. For example, it has been shown that point mutations in the *P. falciparum *dihydrofolate reductase (*dhfr*) gene at codons A16V, N51I, C59R, S108N/T and I164L augmented by mutations in the dihydropteroate synthase (*dhps*) gene at codons S436F, A437G, K540E, A581G, and A613S/T confer resistance to sulphadoxine-pyrimethamine [12-14]. Chloroquine resistance is attributed to SNPs in chloroquine resistance transporter (*crt*) and/or multidrug resistance gene 1 (*mdr1*) genes [15-18].

Most of the methods used for detection of drug resistance SNPs are polymerase chain reaction (PCR) based. Once amplicons are obtained, the presence of SNPs can be detected by restriction fragment length polymorphisms (RFLP), DNA sequencing, or single-stranded conformation polymorphisms [19]. Other methods used for SNP analysis include single-nucleotide primer extension (SNPE) [20], melting curve analysis-fluorescence resonance energy transfer (FRET-MCA) [21], molecular beacons [22], melting probe hybridization [23], real-time PCR [24] and microarray [25]. Each of these methods presents unique advantages and disadvantages. While there is a need to develop new SNP analysis methods it is important that next generation SNP analysis improves on the existing methods. Some desirable qualities for next generation SNP analysis methods should include improved accuracy and sensitivity, reduced cost, fast turn-around time, high through-put capability, and field deployable to mention a few.

Here, development of TaqMan Allelic Discrimination assays (SNP assays) for genotyping SNPs associated with *P. falciparum *drug resistance performed on Applied Biosystems 7500 Fast Real-Time PCR System is described. The performance of each SNP assay was assessed using plasmid DNAs which contained PCR fragment carrying codons of interest from *P. falciparum *laboratory strains with known genetic profile. The specificity of the SNP assays was assessed by comparing SNP assay data to sequence data of clinical samples.

## Methods

### Clinical samples

Samples used in this study were obtained from a Phase IIb paediatric clinical trial conducted between March 2005 and April 2006 at the KEMRI/Walter Reed Project, Kombewa Clinic in the Kombewa Division of Kisumu District, Nyanza Province, Western Kenya. The study was approved by the Ethical Review Committee of the Kenya Medical Research Institute, Nairobi, Kenya and Walter Reed Army Institute of Research (WRAIR) Institute Review Board, Maryland, USA. The details of this study have been described elsewhere [26]. Genomic DNA from the clinical sample was extracted from whole blood using the QIAamp DNA Blood Mini Kit (Qiagen, Valenica CA, USA) as recommended by the manufacturer.

### *Plasmodium falciparum *laboratory strain samples

Genomic DNA from three *P. falciparum *laboratory strains was used in this study. Genomic DNA from 3D7 and 7G8 strains was extracted from cultures grown at WRAIR using QIAamp DNA Blood Mini Kit. K1strain genomic DNA was obtained from Malaria Reagent Repository Resource http://mr4.org, MR4.

### SNP selection and assay design

Thirty SNP sequences (~100 nucleotides on the left frank and ~100 nucleotides on the right frank of the allele of interest) associated with anti-malarial drug resistance were submitted to Applied Biosystems (AB) website for analysis of their suitability to be used in SNP assays. After submitting sequences to the custom TaqMan assay design tool on the AB website, twenty five sequences returned SNP assays that were considered to be good candidate designs. The following SNP assays were selected for further analysis: *pfdhfr *codons A16V, S22N, N51I, C59R, S108N, and I164L; *pfmdr1 *codons N86Y, Y184F, S1034C and N1042D; and *pfdhps *codon A581G. TaqMan-MGB genotyping assay mix for each of these assays was ordered from AB. Each of these TaqMan-MGB genotyping assay mix contained a forward and reverse primer, one probe that perfectly matched to the wild-type sequence variant labeled with VIC and the second probe matched to the mutant (SNP) sequence variant labeled with 6-carboxyfluorescein (FAM) (Table 1). Probes contained a non-fluorescent quencher and a minor groove binder moiety (MGB) which allows for shorter probe sequences to be designed. TaqMan-MGB genotyping assay mixes were supplied at 40X concentration.

**Table 1:** Primers and probe sequences of the SNP assays.

SNP Assay Name	Primers/Probes	Sequence (5'-3')
DHFR16	Forward	CAAGTCTGCGACGTTTTCGATATTT
	Reverse	CCTCATTTTTTTTCCCCTCATTTTTGC
	Probe1	CCTTACAACATGCACATAT
	Probe2	AACCTTACAACATACACATAT

DHFR22	Forward	GCGACGTTTTCGATATTTATGCCATA
	Reverse	TCCTAGACCTCTAAATGTGTAGTTATTAAAAACCT
	Probe1	CCCTCATTTTTGCTTTCAA
	Probe2	CCCTCATTTTTGTTTTCAA

DHFR51	Forward	ACTACACATTTAGAGGTCTAGGAAATAAAGGA
	Reverse	GTTGTAACTGCACAAAAATATTTCATATCTAGGG
	Probe1	CCATGGAAATGTAATACCAT
	Probe2	CCATGGAAATGTATTACCAT

DHFR59	Forward	CTAGGAAATAAAGGAGTATTACCATGGAAATGT
	Reverse	CATCTCTTATATTTCAATTTTTCATATTTTGATTCATTCA
	Probe1	CCCTCATTTTTGCTTTCAA
	Probe2	CCCTCATTTTTGTTTTCAA

DHFR108	Forward	ATGTAAATGATATGCCTAATTCTAAAAAATTACAAAATGT
	Reverse	GACAATATAACATTTATCCTATTGCTTAAAGGT
	Probe1	CTTTCCCAGCTTGTTCT
	Probe2	CTTTCCCAGTTTGTTCT

DHFR164	Forward	ACAAAGTTGAAGATCTAATAGTTTTACTTGGGAAA
	Reverse	TTAATTTCTTTTCTAAAAATTCTTGATAAACAACG
	Probe1	CCCTCATTTTTGCTTTCAA
	Probe2	CCCTCATTTTTGTTTTCAA

MDR86	Forward	AGGAGGAACATTACCTTTTTTTATATCTGTGT
	Reverse	ATTGTACTAAACCTATAGATACTAATGATAATATTATAGGAT
	Probe1	CATCACCTAAATTCATGTTC
	Probe2	CATCACCTAAATACATGTTC

MDR184	Forward	GGTACGAAATTTATAACAATTTTTACATATGCCAGTT
	Reverse	AAAAACGCAAGTAATACATAAAGTCAAACGT
	Probe1	CCTTTTTAGGTTTATATATTTG
	Probe2	CCTTTTTAGGTTTATTTATTTG

MDR1034	Forward	GACAAAAAAGAAGAATTATTGTAAATGCAGCTT
	Reverse	AGGATCCAAACCAATAGGCAAAACT
	Probe1	CTTTGACTGAATCCC
	Probe2	TTTGACAGAATCCC

MDR1042	Forward	GGATTCAGTCAAAGCGCTCAATTA
	Reverse	GTACCTCTTTTAATTAAGAAGGATCCAAACCA
	Probe1	ATAGGCAAAACTATTAATAAA
	Probe2	TAGGCAAAACTATCAATAAA

DHPS581	Forward	TTCTTGTATTAAATGGAATACCTCGTTATAGGAT
	Reverse	TATACATGTATATTTTGTAAGAGTTTAATAGATTGATCATG
	Probe1	TTTCTTCGCAAATCC
	Probe2	TTTCTTCCCAAATCC

### SNP assay

A working master mix was prepared that contained 0.25 μL of TaqMan-MGB genotyping assay mix (20X), 2.5 μL TaqMan Genotyping Master Mix (Applied Biosystems, Carlsbad CA, USA) and 2.25 μL of water or 1.25 μL water and 1 μL of human genomic DNA at 50 ng. Human genomic DNA was added in reactions which were ran with plasmid DNAs as the template. Each reaction contained 5 μL of working master mix and up to 2 μL of template DNA. Template DNA used in these experiments was either plasmid DNA or genomic DNA. In some experiments, two different plasmid DNAs were used. In such instances, 0.5 μL or 1 μL of each plasmid DNA was added to the 5 μL of the working master mix bringing the final volume of the reaction to 6 or 7 μL. Extensive experiments (to be reported elsewhere) have been done which show that such differences in volume and concentration of real-time PCR reactions do not alter assay performance. Plates were loaded into Applied Biosystems 7500 Fast Real-Time PCR System and AB 7500 v 2.0.5 software was used to run the genotyping assay experiment following the default standard Allelic Discrimination genotyping assay protocol. The initial step of this protocol includes pre-reading of the plate where the background florescence is recorded followed by AB standard PCR protocol of 95°C for 10 min, 95°C for 15 sec and 60°C for 1 min, repeating steps 2-3 for 40 cycles. The post-read step follows after completion of the PCR step where a post-read is performed. The software analysed the before and after florescence level and calculates normalized dye fluorescence (ΔRn) as a function of cycle number for Allele1 (wild-type) or Allele2 (mutant). Based on this number (which is a relative number of the possible outcomes), the software makes an automatic call of either Allele1 (homozygous1/1), Allele2 (homozygous2/2) or heterozygous (1/2). In some instances however, the software does not make automatic calls and returns a call of undetermined which can be due to low or no change of fluorescence. Also, the system requires large enough sample number in order to make automatic calls (from communicating with AB technical help line, ~fifteen or more samples are required for the software to make automatic call). However, in some instances there is no clear explanation why the software does not make automatic calls but with proper controls in place, calls can be made manually by assessing the calculated normalized dye fluorescence (ΔRn) as a function of cycle number and the cycle threshold (CT) values.

### DNA amplification and cloning PCR fragments into TOPO TA vector

Primers were designed (using Primer Express 3.0 software) that amplified amplicons of interest (Table 2). DNA was amplified from genomic DNA of either *P. falciparum *laboratory strain samples or clinical samples. The amplification reaction mixture contained 1 × PCR gold buffer with MgCl_2 _in a final concentration of 4 mM, 0.2 mM deoxynucleoside triphosphates, and 0.4 μM of each primer. Reactions were carried out in 20 μL reactions containing 1 μL genomic DNA and 1 U AmpliTaq Gold DNA Polymerase with GeneAmp (Applied Biosystems, Carlsbad CA, USA). Cycling conditions were 95°C for 10 min followed by 40 cycles of 95°C for 15s, 52°C for 10s, and 72°C for 30s. Amplification of the PCR fragments was confirmed by running part of the PCR reaction on a 2% agarose gel and staining with ethidium bromide. PCR fragments were purified using QIAquick PCR purification kit (Qiagen, Valenica CA, USA) following manufacturer's instructions. Purified PCR fragments were cloned into pCR 2.1-TOPO vector using TOPO TA cloning kit (Invitrogen, USA) following manufacturer's recommendations. One Shot TOP10 Chemically or Electrocomp competent cells (Invitrogen, USA) where transformed with cloned vector following manufacturer's recommendations. These reactions were plated out on kanamycin plates and single colonies were grown in cultures overnight. Plasmid DNAs were purified from overnight cultures using QIAgen spin miniprep kit (Qiagen, Valenica CA, USA) following manufacturer's recommendations. Plasmid DNAs were purified from a minimum of three colonies for each reaction. To verify if cloning reaction was successful, PCR was performed using 1 μL of plasmid DNA as template and primers corresponding to those used to amplify the PCR fragment cloned into the vector. PCR fragments were analysed on agarose gel, ensuring the correct PCR fragments were obtained. Clones with correct PCR fragments were further confirmed by sequencing as described below. The concentration and purity of purified plasmid DNA was measured using NanoDrop 2000 (Thermo Fisher Scientific Inc, USA) following manufacturer's instructions. All DNA samples were required to have a 260/280 ratio of between 1.8 and 2.2. Most samples had 260/280 ratio of between 1.88 and 2.0. Plasmid DNA Genomic Equivalence (GE) was calculated using the following equation:

**Table 2:** Primer sequences used in generation of plasmid DNAs.

Plasmid generated		Sequence (5'-3')	PCR fragment size
16-3D7/7G8	Forward Primer	CAAGTCTGCGACGTTTTCGATATTT	
	Reverse Primer	TTAATTTCTTTTCTAAAAATTCTTGATAAACAACG	526 bp

86-K1/7G8	Forward Primer	TGGGTAAAGAGCAGAAAGAGAAA	
	Reverse Primer	TTGCAACAGTTCTTATTCCCATT	735 bp

1034-K1/7G8	Forward Primer	CAAGCGGAGTTTTTGCATTT	
	Reverse Primer	TTCCACCATCATCTCTTACATCA	447 bp

581-K1/7G8	Forward Primer	TGCATAAAAGAGGAAATCCACA	
	Reverse Primer	TCCAATTGTGTGATTTGTCCA	357 bp

$$\left(\text{X g}/\mu \text{L DNA}/\left[\text{transcript length nucleotide}\times \text{66}0\right]\right)\times \text{6}.0\text{22}\times \text{1}0^{\text{23}}=\text{Y molecule}/\mu \text{L}$$

### Sequencing

Bi-directional sequencing was performed either on plasmid DNA or directly on PCR fragments obtained from genomic DNA. DNA amplifications were performed as described above and PCR fragments purified using QIAquick PCR purification kit. BigDye Terminator v3.1 Cycle Sequencing Kit (Applied Biosystems, Carlsbad CA, USA) was used for bi-directional sequencing following manufacturer's recommendations. Each reaction contained 4 μL of Terminator Ready Reaction Mix, 3.2 pmol primer, template DNA (plasmid DNA or purified PCR fragment) in a final volume of 10 μL. M13F or M13R primers which are part of TOPO TA cloning kit were used for the bi-directional sequencing of plasmid DNAs whereas forward or reverse primers used in generation of the PCR fragments were used in sequencing of these amplicons. Cycling conditions for the BigDye Terminator reaction was as follows: 96°C for 1 min followed by 25 cycles of 96°C for 10s, 50°C for 5s, and 60°C for 4 min. The products of these reactions were purified using Performa Spin Columns (EdgeBio, Gaithersburg MD, USA) following manufacturer's recommendation. Electrophoresis of these samples was performed using 3130XL Genetic Analyzer (Applied Biosystems, Carlsbad CA, USA) following manufacturer's recommendation.

## Results

### SNP assays performance

**Figure 1:**
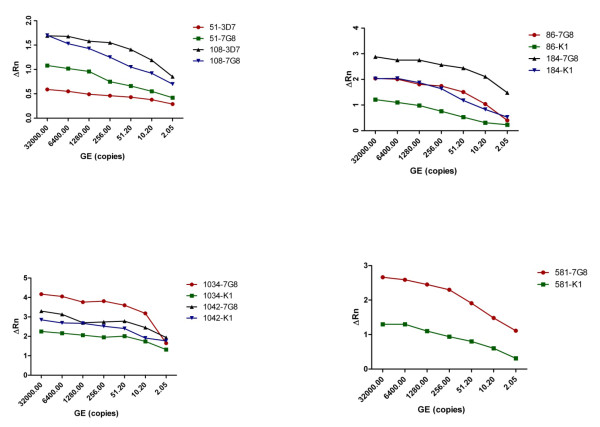
**LoD for DHFR51 and DHFR108, MDR86 and MDR184, MDR1034 and MDR1042, DHPS581 SNP assays**. Data showing LoD for allele1 and allele2 for each SNP assay where plasmid DNA carrying either allele1 or allele2 was used as a template. LoD for all the SNP assays was established at 2 GE. Data is shown indicating the SNP assay and the strain which the PCR fragment in the plasmid DNA was cloned from. Example 51-3D7 is data obtained from SNP assay DHFR51 using 16-3D7 plasmid DNA.

To analyse the performance of the selected SNP assays, three *P. falciparum *strains, 3D7, 7G8 and K1 that differ in some of their genetic profiles at selected codons (Table 3) were used to generate plasmid DNAs containing PCR fragment of genes carrying codons of interest. Four PCR fragments that contained all the eleven SNPs were amplified from respective genes and cloned into TOPO TA vectors as described in the methods section. Primers used for amplification of these PCR fragments are shown on table 2. Amplicon sizes were as follows; the amplicon that carried *pfdhfr *codons A16V, S22N, N51I, C59R, S108N, and I164L was 526 bp; the amplicon that carried *pfmdr1 *codons N86Y and Y184F was 735 bp; the amplicon that carried *pfmdr1 *codons S1034C and N1042D was 447 bp; and the amplicon that carried *pfdhps *codons A581G was 357 bp. *Pfdhfr *codons A16V, S22N, N51I, C59R, S108N and I164L PCR fragments were amplified from 3D7 and 7G8 strains. These fragments were cloned into TOPO TA vectors and the plasmid DNAs generated are referred to as 16-3D7 and 16-7G8. Plasmid DNA 16-3D7 was used for analysis of codons A16V, S22N, C59R, and I164L. Note that the genetic profiles of these codons are the same (wild-type) in 3D7 and 7G8 strains. Plasmid DNAs 16-3D7 and 16-7G8 were used for analysis of codons N51I and S108N, which 3D7 and 7G8 strains have different genetic profiles at these codons. For codons *pfmdr1 *N86Y, Y184, S1034C, N1042D and *pfdhps *codon A581G, plasmid DNAs were generated by cloning PCR fragments from K1 and 7G8 strains. K1 and 7G8 strains have different genetic profiles at these codons. The generated plasmid DNAs are referred to as follows; plasmid DNAs carrying *pfmdr1 *codons N86Y or Y184F are referred as 86-K1 or 86-7G8; plasmid DNAs carrying *pfmdr1 *codons S1034C and N1042D are referred to as 1034-K1 or 1034-7G8; and plasmid DNAs carrying *pfdhps *codon A581G are referred to as 581-K1 or 581-7G8. The concentration of each plasmid DNA was determined using NanoDrop and the genomic equivalence (GE) was calculated. To obtain the initial analytical sensitivity and specificity, each plasmid DNA was diluted to an initial final concentration of 32, 000 copies. SNP assays were performed as described in methods section. SNP assay names were based on the codon being analysed. For example, to analyse codon *pfdhfr *N51I, the SNP Assay name is referenced as DHFR51 (Table 1). Performance of each SNP assay was assessed based on the known genetic profile of each strain at each codon and sequence data. Assessment of each SNP assay was based on automatic calls, normalized dye fluorescence (ΔRn) as a function of cycle number for Allele1 (wild-type) or Allele2 (mutant), and the CT values. All the calls made (11 of 11) for each SNP assay were correct, establishing the initial analytical sensitivity and specificity at 100%. To establish the Limit of Detection (LoD) for each assay, each plasmid DNA was serially diluted 5-fold and the initial tentative LoD for each assay was set as the lowest concentration of DNA that yielded positive results in duplicate experiments (Figure 1: shows LoD for *pfdhfr *51 and 108, *pfmdr1 *86 and 184, *pfmdr1 *1034 and 1042, *pfdhps *581). The LoD was confirmed by testing the LoD concentration in replicates of two or more. All the assays had LoD of 2 GE.

**Table 3:** Genetic profile of *P. falciparum *laboratory strains.

Codon	16	22	51	59	108	164	86	184	1034	1042	581
Wild-type	C	G	A	T	G	A	A	A	A	A	C

Mutant	**T**	**A**	**T**	**C**	**A**	**T**	**T**	**T**	**T**	**G**	**G**

3D7	C	G	A	T	G	A	A	A	A	A	C

7G8	C	G	**T**	T	**A**	A	A	**T**	**T**	**G**	C

K1	C	G	N	N	**A**	A	**T**	A	A	A	**G**

Genetic profile of *P. falciparum *laboratory strains used in this study based on PlasmoDB website www.plasmodb.org and/or sequence data. Bold indicates the mutant allele and N indicates there is no information available on the profile of the indicated allele on PlasmoDB website.

### Mixed infections

**Figure 2:**
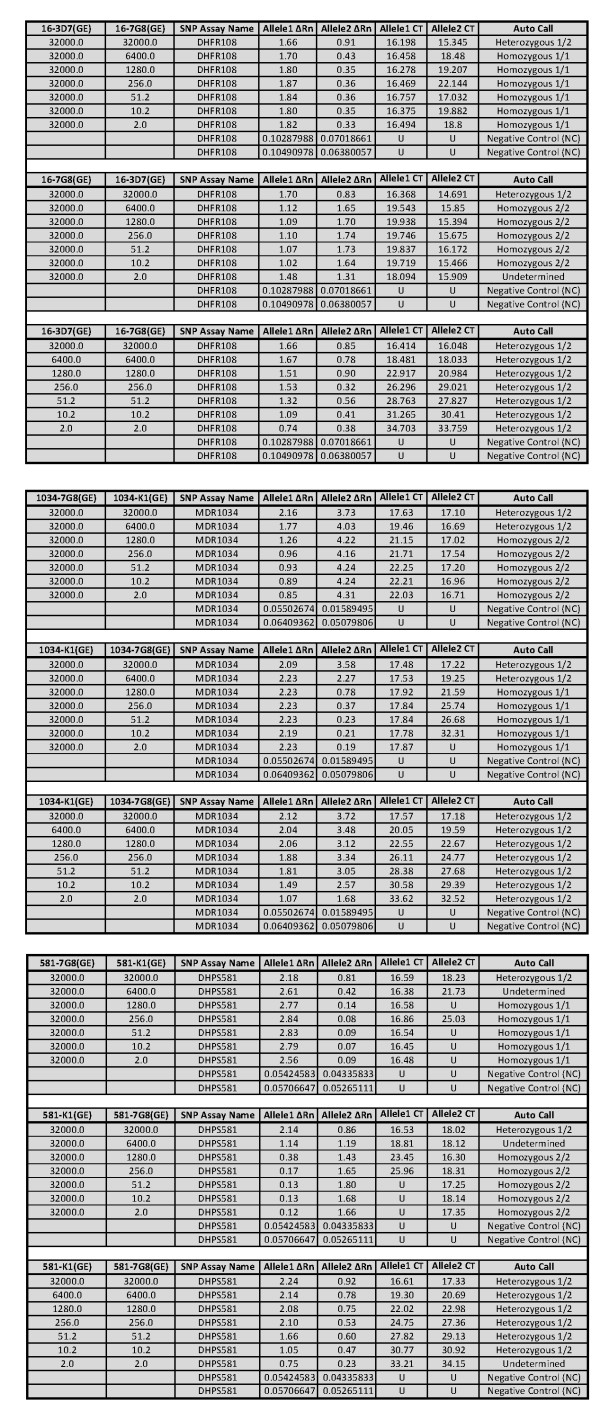
**Performance of DHFR108, MDR1034 and DHPS581 SNP assays in mixed infection experiments**. Plasmid DNAs carrying one of the alleles for each SNP assay were used in these experiments by mixing them in a each reaction. Plasmid DNAs were serially diluted 5-fold starting at 32000 GE down to 2 GE. In some experiments, the concentration of one of the plasmid DNA was kept constant while the other was serially diluted whereas in other experiments, both plasmid DNAs were serially diluted and mixed at equal DNA concentrations.

**Figure 3:**
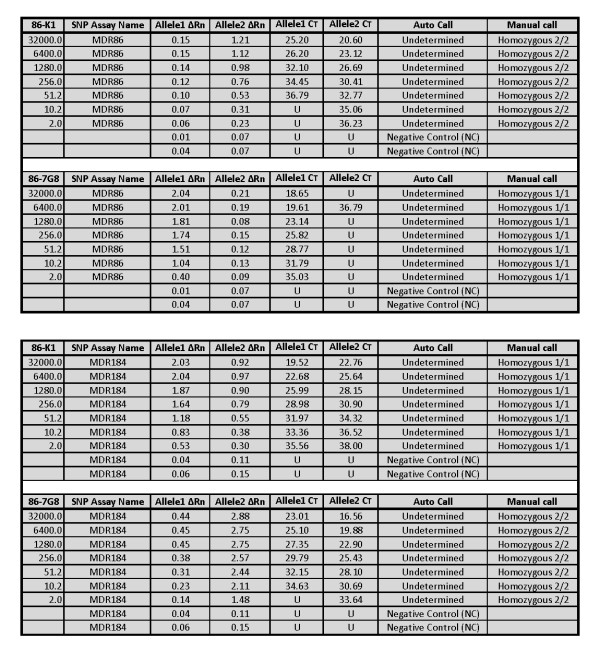
**Manually called SNP assays**. Data showing the manual call of MDR86 and MDR184 SNP assays. The automatic call for these SNP assays during these runs were undetermined however as the data shows, Allele1 and Allele2 ΔRn in these SNP assays were clearly separated. This data shows manual calls can be made with great confidence regardless of whether automatic calls are made or not. In addition, CT values showed a clear and distinct difference between the two alleles.

To test the ability of each assay to discriminate mixed infections, plasmid DNAs carrying alternate genetic profiles at each codon were mixed at different ratios and the ability of each assay to make the correct call was assessed. SNP assays analysed for mixed infection were DHFR51 and 108 (using plasmid DNA 16-3D7 and 16-7G8), MDR86 and 184 (using plasmid DNA 86-K1 and 86-7G8), MDR1034 and 1042 (using plasmid DNA 1034-K1 and 1034-7G8), and DHPS581 (using plasmid DNA 581-K1 and 581-7G8). Analysis for mixed infection was performed as follows; one plasmid DNA concentration was kept constant while the other was serially diluted 5-fold or both plasmid DNAs were serially diluted 5-fold and mixed at equal concentrations. As shown in Figure 2 (DHFR108 SNP assay, MDR1034 SNP assay and DHPS581 SNP assay), when one plasmid DNA concentration was kept constant (high) while the other was serially diluted, the presence of heterozygous alleles was only detected when both plasmid DNAs were at high concentration. The presence of the allele in the serially diluted plasmid DNA was not detected beyond when the plasmid DNAs were at equal concentrations. However, for MDR1034 SNP assay, both alleles were detected when one plasmid DNA was diluted to 6400 GE (Figure 2, MDR1034 SNP assay) while the other was at kept at 32000 GE. Curiously, the software did not make automatic calls for the DNA mixtures that contained serially diluted plasmid DNA at 1:5 (6400 GE) and undiluted plasmid DNA (32000 GE) for DHFR51, MDR1042 and DHPS581 SNP assays, rendering these six assays undetermined. There is no clear explanation why such results were obtained (undetermined) at these plasmid DNA mixture ratios since the software made automatic calls for the consequent plasmid DNA mixtures. Note that the software did not make automatic calls for all MDR86 and MDR184 SNP assays. Notwithstanding, Allele1 and Allele2 ΔRn in these SNP assays were clearly separated but yet the software could not make automatic calls (Figure 3). Manual calls were made based on ΔRn values of the Allele1 and Allele2. It was determined the performance of these two SNP assays, MDR86 and MDR184 were similar to those of the other SNP assays. Interestingly, in all the SNP assays, when both plasmid DNAs were serially diluted, the presence of both alleles was detected at or near LoD as established using single plasmid DNA.

### Assay specificity

**Figure 4:**
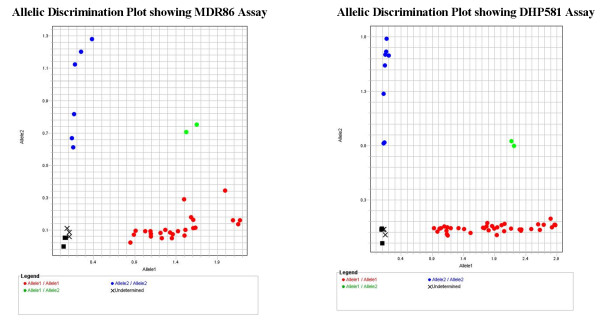
**Allelic Discrimination plots for MDR86 and DHPS581 SNP assays**. Two representative plots showing performance of two assays in analysis of clinical samples. These assays show clear separation between the signals derived from allele1 or allele2. Allele1 is shown in red and allele2 is shown in blue. Lime green represents a mixture of the two alleles (which was a positive control allele1/allele2 derived using a mixture of two plasmid DNAs).

Twelve samples from the clinical trial were randomly selected and sequenced directly or by cloning PCR fragment that contains codons of interest into TOPO TA vector. Primers used for cloning and/or sequencing are described in Table 2. Here, similar scheme was used as that described in the results section, SNP assay performance. PCR fragments were amplified using different primer sets and amplicons were either cloned into TOPO TA vector and then sequenced or were directly sequenced as described in the methods section. SNP assays were performed in replicates of two or more for each sample using either cloned plasmid DNA and/or genomic DNA of the clinical samples. 3D7, 7G8 or K1 plasmid DNAs were used as positive controls for each allele in all the assays analysed. For DHFR16, 22, 59, and 164 SNP assays, only 16-3D7 plasmid DNA was used as positive control (Allele1) because at these genetic loci, both 3D7 and 7G8 strains carry wild-type alleles. For DHFR51 and 108 SNP assays, 16-3D7 and 16-7G8 plasmid DNAs were used as positive controls (Allele1 and Allele2 respectively) because at these genetic loci, 3D7 and 7G8 carry different alleles (Table 3). 86-7G8 and 86-K1 plasmid DNAs were used as Allele1 and Allele2 positive controls respectively for MDR86 SNP assays whereas for MDR184 SNP assays, 86-K1 and 86-7G8 plasmid DNAs were used as Allele1 and Allele2 positive control respectively. 1034-K1 and 1034-7G8 plasmid DNAs were used as Allele1 and Allele2 positive controls for MDR1034 and 1042 SNP assays respectively. For DHPS581 SNP assay, 581-7G8 and 581-K1 plasmid DNAs were used as Allele1 and Allele2 positive controls respectively. K1 and 7G8 strains differ at all these genetic loci as shown (Table 3). SNP assays were performed as described in the methods section. Figure 4 shows an Allelic Discrimination plots for DHPS581 and MDR86 SNP assays ran using clinical samples and plasmid DNA controls. The plot shows a clear and distinct separation between the two alleles. In these experiments unlike those initially ran, automatic calls were made for MDR86 and 184 SNPs assays since large number of samples and controls were used, emphasizing the need of analyzing a large number of samples for the software to make automatic call. To assess the analytical specificity of the SNP assays, calls made using SNP assay data were compared to the sequence data of the samples. All calls made using SNP assays were in 100% agreement with sequence data. Sequence analysis is considered a gold standard method for SNP analysis. Eleven SNP assays were analysed in 12 clinical samples, representing a total of 132 SNP assays that were correctly called. This analysis, in addition to that initially performed using 3D7, 7G8 and K1 strains represent 100% specificity of all the SNP assays analysed.

### Clinical samples analysis

Sixty clinical samples with known parasite density based on microscopy and real-time PCR data [27] were analysed using all the eleven SNP assays. All the SNP assays performed as expected with distinct and clear calls regardless of the parasite density in each sample. In additional, 10 clinical samples that were negative by microscopy and real-time PCR data were ran for further analysis of the analytical specificity of the SNP assays. There were no false positives; all the CT values were undetermined.

## Discussion

A TaqMan Allelic Discrimination assays for detection of SNPs associated with anti-malarial drug resistance has been described. The assay was developed for use on Applied Biosystems 7500 Fast Real-Time PCR System. The performance of each of the SNP assays was analysed and control plasmid DNAs developed. Each SNP assay performed well with 100% sensitivity and specificity. Data from genetic profiles of the *P. falciparum *laboratory strains and sequence data from 12 clinical samples was used as the reference method with which the SNP assays were compared to. All the SNP assays had LoD of 2 copies, which is equivalent to less than 1 parasite/μL (based on unpublished data to be reported elsewhere). The performance of the SNP assays was further validated when each SNP assays was used to assess SNP genetic profile of additional 60 clinical samples with known parasite density based on microscopy and real-time PCR assay which amplifies 18s RNA. Based on microscopy, the density of the parasite ranged from less than a 100 parasites/μL to more than 10E6 parasites/μL [27]. All SNP assays performed well regardless of parasite density. Using real-time PCR 18s RNA parasite density data, SNP assays successfully genotyped samples containing parasites densities of 10 parasites/μL or less. All clinical samples that were negative by real-time PCR 18s RNA assay were also negative in all SNP assays.

Detection of SNPs associated with anti-malarial drugs using real-time PCR based assay has been extensively reported [28]. However, until recently [29], there has not been any published work towards developing SNP detection assays using the TaqMan Allelic Discrimination assay method on Applied Biosystems PCR platform for genotyping SNPs present in the *Plasmodium *genome. In the study by Daniel *et al*., [29], TaqMan Allelic Discrimination assay was developed for identification and tracking *P. falciparum*. Here, the authors selected a panel of twenty-four SNPs that were identified to exhibit a high minor allele frequency for which robust TaqMan genotyping assays were constructed. A variety of samples including *P. falciparum *laboratory strains, frozen whole blood, or whole blood spotted onto filter paper were used in the assay with a success rate > 99%. The performance of the assay was validated using nested PCR. By making calls using ΔRn, TaqMan Allelic Discrimination assays simplifies and increases accuracy in call making by eliminating subjective decision which can be made by use of CT values. In most of the mixed infections, data present here show that if only CT values were used, wrong calls would have been made. For example in Figure 3 where 1034-K1 plasmid DNA at 32000 GE was mixed with 1034-7G8 plasmid at 6400 GE, the MDR1034 SNP assay had CT values of 17.53 and 19.25 for Allele1 and Allele2 respectively and ΔRn values of 2.23 and 2.27 for Allele1 and Allele2 respectively. Here, heterozygous1/2 automatic call was made but if this decision was based on CT values, probably subjective call would have been Allele1. Automatic calls are less subjective since they are based on the software's algorithm. It is important to have positive and negative controls in place and a large number of samples must be analysed for the software to make automatic calls.

Currently, there are many methods that are being used for molecular analysis of anti-malarial drug resistance. Methods chosen by different studies will be based on many factors including technology capabilities and availability, the availability of intellectual and financial resources to mention a few. Some of the factors that should be important in decision making regarding what assay to use in SNP analysis should include accuracy of the assay, sensitivity, robustness, reproducibility, cost, and reliability. Here, we are adding one more tool that can be used in genotyping anti-malarial drug resistance.

TaqMan Allelic Discrimination assays can be custom designed for detection of SNPs of interest. When designing these assays however, just like any other PCR assay, not all SNP sequences will be good candidate designs. And once good candidate designs are identified, it is important that extensive analysis of each assay is performed before its utilization. It is important that a SNP detection assay is capable of detecting mixed alleles (mixed infection). Here, just like previously reported [29], data shows that TaqMan Allelic Discrimination assay is capable of detecting mixed alleles. However, it is important to note that this is a PCR based assay which discriminates the presence of either allele based on affinity of one probe to the SNP sequences of the allele present as opposed to the one not present. Some SNP assays might detect mixed infections better than others; it all depends on chemistry of each set of probes. Data presented here show that SNP assays can detect mixed infections when DNA of both alleles is present in high to very low concentrations as long as DNAs carrying both alleles are at or near equal concentration.

## Conclusion

The SNP assay described here for detection of anti-malarial drug resistance is accurate, sensitive, robust, inexpensive, highly reproducible and reliable. And since it is a real-time PCR based assay, it has great potential of being utilized in high-throughput studies such as surveillance and epidemiological studies. TaqMan Allelic Discrimination assays have been widely developed and used for genotyping SNPs in applications such as SNP typing in forensic genetics, pharmacokinetics and bacterial strain typing [30]. However, it is not until recently that Daniels *et al *[29] showed utilization of this technology in allele discrimination in *Plasmodium *genome. It is important to note however that all real-time PCR based assays will poorly detect mixed infections where one allele is at much higher concentration than the other. If there is need to resolve mixed infection that might have large differences in DNA concentration between the two alleles, other technologies such as nested PCR must be utilized. To alleviate limitation associated with detecting mixed infection, TaqMan Allelic Discrimination assay can be modified by performing nested PCR where flanking primers can be used in first round to improve the likely hood of detecting mixed infections during the real-time PCR second (nested) round.

## Competing interests

The authors declare that they have no competing interests.

## Authors' contributions

EK conceived and designed the study, acquired, analysed and interpreted the data; wrote the first draft of the manuscript. SA performed the sequencing experiments, SNP assay for the clinical samples and reviewed manuscript. KF analysed sequences generated and reviewed manuscript. LT processes cultured samples, run some experiments and reviewed manuscript. BO was the principles investigator the Phase IIb pediatric clinical trial. CFO authorized, supported, contributed intellectually, and reviewed the manuscript.
